# CDR1as modulates arrhythmia post-myocardial infarction via regulating Cav1.2: CDR1as damages the Cav1.2 channel in the heart

**DOI:** 10.3724/abbs.2025126

**Published:** 2025-07-29

**Authors:** Jiapan Wang, Wenjie Liao, Xingda Li, Zhen Chen, Chunlei Duan, Zhenru Wang, Hongda Li, Haonan Du, Ye Yuan, Zhimin Du

**Affiliations:** 1 Institute of Clinical Pharmacy of the Second Affiliated Hospital of Harbin Medical University State Key Laboratory of Frigid Zone Cardiovascular Diseases (SKLFZCD) Harbin 150001 China; 2 State Key Laboratory of Quality Research in Chinese Medicines Macau University of Science and Technology Macau 999078 China

**Keywords:** myocardial infarction, ventricular arrhythmia, CDR1as, Cav1.2, *I*_CaL_

## Abstract

Arrhythmias, especially ventricular arrhythmias (VAs), are the primary cause of mortality following myocardial infarction (MI) and are typically attributable to electrophysiological disorders of the heart. Our previous work demonstrated that *CDR1as* knockdown ameliorates arrhythmias by modulating Nav1.5 and Kir6.2 channels post-MI. This study aims to explore the role of CDR1as in calcium channel remodeling subsequent to ischemic arrhythmia. We employ MI in mice by ligating the left anterior descending coronary artery (LAD) and use patch-clamp techniques to measure the Ca current ( *I*_CaL_) in isolated ventricular cardiomyocytes. The results show that the expression of Cav1.2 is significantly decreased in the infarct border zone at 12 h post-MI. *CDR1as* knockdown via AAV9-CDR1as-shRNA administration leads to an enhancement of cardiac function and a restoration of both *I*_CaL_ density and Cav1.2 expression in MI model mice. These findings indicate that targeting the CDR1as pathway to modulate calcium channels can be a viable strategy for antiarrhythmic therapy following MI.

## Introduction

Cardiac arrhythmias represent a significant global health burden, contributing substantially to morbidity and mortality [1]. Ventricular arrhythmias (VAs) are of particular concern, as they are the predominant cause of sudden cardiac death in patients who have experienced MI [ 2, 3]. Numerous studies have demonstrated that alterations in VAs susceptibility following MI are predominantly attributed to structural and electrical remodeling of the heart [4]. The complex interplay of electrophysiological abnormalities following MI lays the foundation for the development of life-threatening arrhythmias. A key mechanism implicated in the pathogenesis of VAs post-MI is the dysregulation of calcium cycling, which affects the cardiac action potential (AP) and engenders delayed depolarizations [5].

Circular RNAs (circRNAs) have emerged as a novel class of noncoding RNAs that are generated through a back-splicing process of pre-messenger RNA (pre-mRNA) transcripts and are more stable than long non-coding RNAs (lncRNAs) [6]. CircRNAs are known to perform a variety of functions within the cell. One of the primary functions of circRNAs is their capacity to function as microRNA (miRNA) sponges, which attach to miRNAs and thereby prevent these miRNAs from interacting with their target messenger RNAs (mRNAs). This mechanism can lead to derepression of gene expression and has been implicated in numerous biological processes [ 7, 8]. The investigation of circRNAs in the context of cardiac arrhythmias is particularly intriguing. Ion channel remodeling, which involves the abnormal function or expression of sodium, potassium and calcium ion channels, is the main cause of arrhythmia [9]. Our previous study revealed that knockdown of circRNA *CDR1as* can shorten the duration of the QRS complex and QTc interval, ameliorate electrical remodeling disturbances caused by dysregulation of Nav1.5 and Kir6.2 channels in cardiomyocytes, and reduce susceptibility to VAs [10]. Given the critical role of calcium ions in cardiac electrophysiology, any dysregulation of calcium homeostasis can lead to arrhythmia [ 11– 13]. However, it is unclear whether the association between CDR1as and ischemic arrhythmias is related to calcium ion interference.

The rhythmic contraction of the heart begins with the opening of the L-type Ca ^2+^ channel (LTCC) and the inflow of extracellular Ca ^2+^. Defects in intracellular Ca ^2+^ homeostasis have been identified as key factors in the increased tendency toward arrhythmia in acquired heart disease, such as heart failure (HF) or diabetic cardiomyopathy [14]. Therefore, the intracellular LTCC is essential for cardiac systolic function and electrophysiological balance [ 15, 16].

In this study, our objective was to investigate the potential role of CDR1as in the modulation of the LTCC post-MI and its implications for the development of arrhythmias. We hypothesized that CDR1as contributes to the pathogenesis of VAs by disrupting LTCC function, thereby providing a new potential therapeutic target for the management of arrhythmias after MI. We found that downregulation of CDR1as can improve LTCC channel abnormalities and reduce Cav1.2 and *I*_CaL_ caused by MI. Our study provides compelling evidence that the intervention of LTCC through the CDR1as pathway is a novel, feasible and promising antiarrhythmic approach.

## Materials and Methods

### Animals

Male C57BL/6 mice (20 – 25 g) were purchased from the Laboratory Animal Center of the Second Hospital of Harbin Medical University. The mice were fed under standard animal chamber conditions (temperature 23 ± 1°C, humidity 55%–60%). The experimental mice were randomly assigned to different groups. All experimental animal protocols were approved by the Institutional Animal Care and Use Committee of Harbin Medical University and the Ethics Committee of Harbin Medical University (IRB 3006822). This study complies with guidelines for the Care and Use of Laboratory Animals published by the National Institutes of Health (NIH Publication No. 85 - 23, revised in 1996).

### Infection with AAV9-adeno-associated virus

AAV9-CDR1as and AAV9-null adeno-associated viruses were purchased from Genechem Co., Ltd. (Shanghai, China). AAV9-shCDR1as and AAV9-shNC adeno-associated viruses were purchased from HANbio Biotechnology (Shanghai, China). AAV9 was injected into the tail vein at a dose of 1 × 10 ^11^ viral genomes (vg) per animal. We constructed an animal model of MI or carried out follow-up experimental analysis 4 weeks later.

### Mouse model of MI

The mice were anesthetized with 2,2,2-tribromoethanol at a dosage of 200 mg/kg (Sigma, St Louis, USA). The chest cavity was subsequently opened to expose the heart, and the left anterior descending coronary artery was wrapped with 7/0 nylon sutures to establish a mouse model of MI. The electrocardiogram (ECG) results indicated significant elevation of the S-T segment, confirming the successful establishment of the model.

### Triphenyl-tetrazolium-chloride (TTC) staining

Twelve hours after MI, the hearts of the mice were collected for TTC staining. Four consecutive 1.0-mm-thick slices were subsequently cut. These slices were incubated with a 2% TTC solution (Solarbio, Beijing, China) at 37°C for 30 min. After incubation, photographs were taken, and the resulting images were analyzed via ImageJ software.

### Neonatal mouse primary cardiomyocyte culture

Cardiomyocytes were obtained from C57BL/6 mice aged 1–3 days. The hearts of neonatal mice were first placed in D-Hank’s solution along with 0.25% trypsin and then digested on a shaker at 4°C. After 12 h, the cardiomyocytes were further digested with type II collagenase solution (Gibco, Grand Island, USA). Isolated cardiomyocytes were collected by centrifugation at 190 g/min for 5 min and then incubated with DMEM containing 10% fetal bovine serum for 50 min. Cardiomyocytes were cultured at 37°C in a cell culture incubator containing 5% CO _2_ and 95% O _2_ for 48 h and then used for subsequent experiments.

### Cell infection with adenovirus

The adenoviruses Adv-CDR1as and Adv-Null were synthesized via the GeneChem technique. These adenoviruses, with a final concentration of 1 × 10 ^7^ PFU/mL, were introduced into the NMCMs for 8 h, and the next experiment was performed after 48 h.

### Cell transfection with siRNA

siCDR1as and negative control RNA (siNC) ( Supplementary Table S1) were synthesized by GENERL BIOL (Chuzhou, China). According to the manufacturer’s instructions, the siRNA was transfected via X-tremeGENE transfection reagent (Roche, Basel, Switzerland) for a duration of 6 h. Subsequent experiments were initiated 48 h post transfection.

### Cell counting kit-8 (CCK8) assay

The CCK-8 assay kit (SEVEN, Beijing, China) was used to assess cell viability. NMCMs were seeded into 96-well plates, subjected to respective treatments, followed by the addition of 10 μL CCK-8 solution to each well. The plates were incubated for one hour at 37 °C, and absorbance at 450 nm was subsequently measured using a microplate enzyme reader (TECAN, Männedorf, Switzerland).

### Lactate dehydrogenase (LDH) Assay

LDH release in the culture supernatant of NMCMs was measured using an LDH detection kit (Nanjing Jiancheng Bioengineering Institute, Nanjing, China) according to the manufacturer’s instructions. Absorbance was quantified at 450 nm using a microplate enzyme reader (TECAN, Männedorf, Switzerland).

### Measurement of cardiomyocyte shortening

Isolated adult mouse cardiomyocytes were placed in a bath containing calcium solution, and when they settled steadily to the bottom of the bath, we used 1 Hz electrical pacing to stimulate the cells. At this time, the cardiomyocytes showed stable contraction, and the IonOptix system (IonOptix Co., Westwood, USA) was used to record video images of the cells. Myocardial contractility strength was calculated as follows: (diastolic length–systolic length)/diastolic length × 100%.

### Echocardiographic analysis

Echocardiography was performed on mice anesthetized with 2,2,2-tribromoethanol. Two-dimensional M-mode and three-dimensional Doppler echocardiography were performed via an ultrasound imaging system (VisualSonics, Toronto, Canada) to evaluate cardiac diameter and function.

### Western blot analysis

Purified proteins from NMCMs or cardiac tissue were separated via SDS-PAGE using 7.5% polyacrylamide gel. The protein bands were transferred to nitrocellulose membranes (PALL, New York, USA). The membranes were blocked and incubated overnight with anti-Cav1.2 antibody (YT7807, 1:500; Immunoway, Plano, USA), anti-RyR2 antibody (YT4196, 1:500; Immunoway) and anti-SERCA2a antibody (27311-1-AP, 1:500; Proteintech, Wuhan, China) at 4°C. The membranes were subsequently incubated with secondary antibodies (Jackson Immuno Research, West Grove, USA). After visualization, images were captured with an Odyssey CLx infrared imaging system (LICOR Biosciences, Lincoln, USA).

### RNA isolation and real-time PCR

NMCM and heart tissue RNA were extracted with TRIzol™ Reagent (Invitrogen; Carlsbad, USA). The concentration and mass of the RNA samples were measured via a NanoDrop ND-2000 (Thermo Fisher Scientific, Waltham, USA). The RNA was reverse-transcribed into cDNA via a reverse transcription kit (Toyobo, Osaka, Japan). The mRNA level of *CACNA1C* was quantified via qRT-PCR via SYBR Green (Roche Holdings AG, Basel, Switzerland).

### Isolation and culture of adult mouse cardiomyocytes

The hearts were quickly removed from the adult mouse, and the aorta was incubated in a constant-flow Langendorff device. A calcium-free solution was injected into the aorta to arrest the heart, and Tyrode’s solution containing 1 mg/mL type II collagenase powder and 0.75 mg/mL bovine serum albumin (Solarbio) was then infused into the digestive heart. After tissue digestion was completed, the infarct marginal heart cells were preserved with Tyrode’s solution containing 200 μM CaCl _2_ and 1% bovine serum albumin.

### Whole-cell patch clamp experiments

Whole-cell patch-clamp experiments were performed via an Axopatch 700B amplifier (Axon instrument, California, USA). L-type calcium currents ( *I*_CaL_) in adult mouse cardiomyocytes were recorded via the whole-cell patch-clamp technique. A depolarization voltage between –60 mV and +60 mV in 10-mV increments causes an L-type Ca ^2+^ current. The external solution for *I*_CaL_ recording contains: 120 mM TEACl, 10 mM HEPES, 1 mM MgCl _2_, 10 mM CsCl, 10 mM glucose and 1.8 mM CaCl _2_, with pH adjusted to 7.35 using CsOH. The pipette solution contained 120 mM CsCl, 40 mM CsOH, 1 mM MgCl _2_, 11 mM EGTA, 5 mM Mg-ATP and 10 mM HEPES, with pH adjusted to 7.3 using HCl. The current amplitude data of each cell are normalized to the cell capacitance (current density, pA/pF), and the current-voltage relationship (I-V curve) is plotted.

### Immunofluorescence staining

Fetal bovine serum was used to block the cultured NMCMs. NMCMs were incubated with anti-Cav1.2 antibody (MA5-27717, 1:300; Invitrogen) mixed with an anti-α-Actinin antibody (GTx29465, 1:300; GeneTex, San Antonio, USA) overnight at 4°C. The cells were washed and incubated with secondary antibodies conjugated to Alexa Fluor 488 (ab 150077, 1:300; Abcam, Cambridge, UK) and Alexa Fluor 594 (ab 150080, 1:300; Abcam) for 1 h at room temperature. The fluorescence intensity of the cells was subsequently measured via a confocal microscope (Zeiss Microsystems, Jena, Germany).

### Statistical analysis

The data are presented as the mean ± SEM. Comparisons among three or more groups were conducted via one-way ANOVA with Tukey’s post hoc test. Statistical significance was defined as a *P* value less than 0.05. The original data points were fitted with a nonlinear least squares curve via GraphPad Prism 9.0.

## Results

### *CDR1as* knockdown can repair the defect of Cav1.2 channel after MI

Cardiac APs involve complex ion transport via Na ^+^, K ^+^ and Ca ^2+^ channels. The Nav1.5 channel controls phase 0 depolarization, whereas the K ^+^ and Ca ^2+^ channels shape the phase 2 plateau. K ^+^ channels also manage phases 3 and 4 (resting the membrane potential). Disruptions in these channels can disturb the balance of ionic currents, causing slow conduction and prolonged action potential duration (APD), as observed in HF and cardiomyopathic models [ 17, 18]. Research has shown that the downregulation of CDR1as can decrease the incidence of ischemic arrhythmias by ameliorating the ischemia-induced reduction in Nav1.5 and Kir6.2 channels [10]. However, the association between CDR1as and Ca ^2+^ homeostasis, particularly in the context of Cav1.2 channels, has not been fully explored. Intracellular calcium ions, which originate from the activation of L-type voltage-gated Cav1.2 channels, are essential for triggering the influx of extracellular calcium ions into the cell, which is a critical process in cardiac electrophysiology [19].

**Figure 1:**
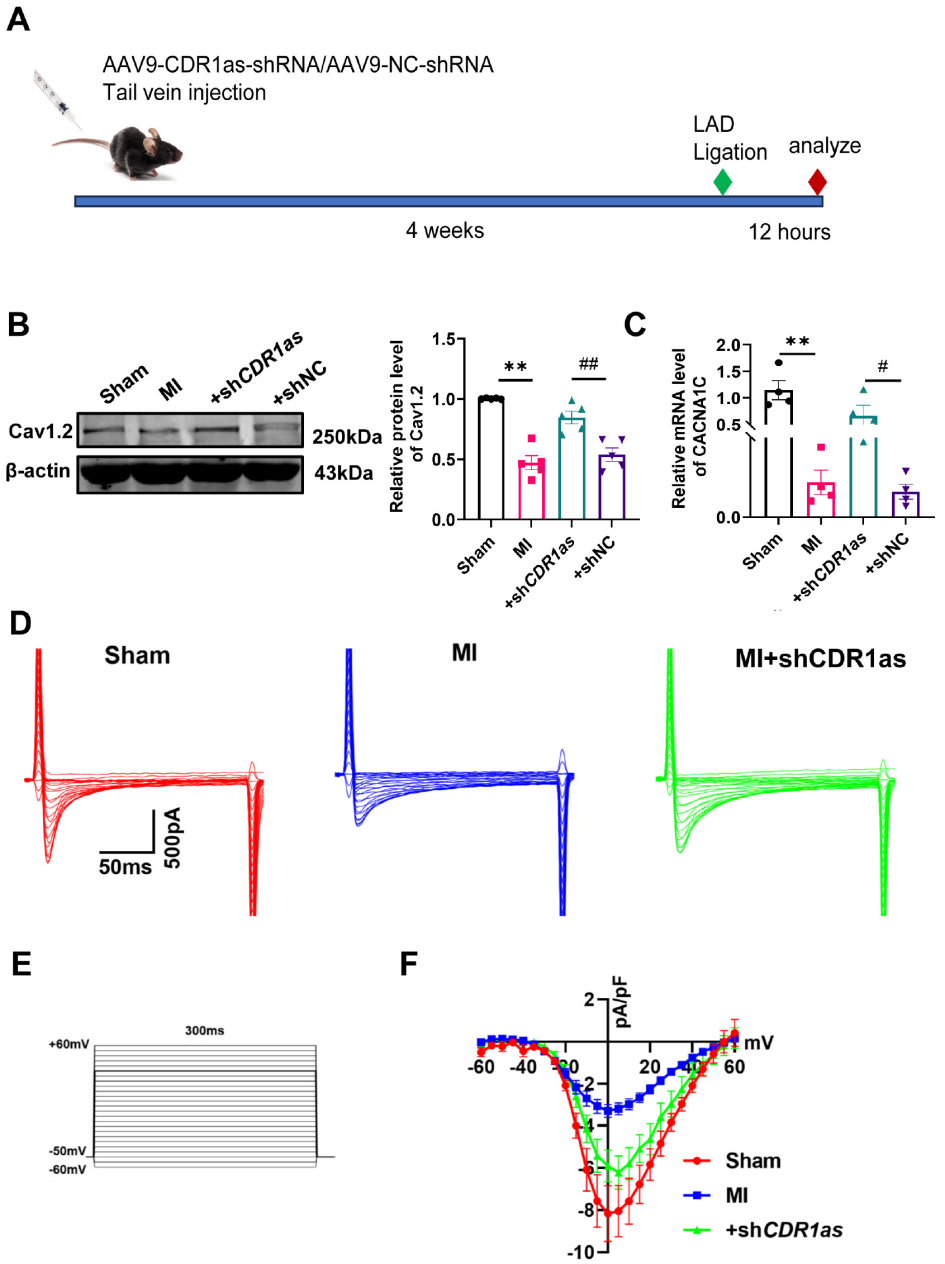
Figure 1 Cav1.2 expression and the *I*_CaL_ are increased in the ventricular myocytes of *CDR1as*-knockdown mice (A) Experimental procedures for AAV9 injection and examinations (MI + AAV9-shCDR1as, MI + AAV9-shNC). (B) Western blot analysis of the protein expression level of Cav1.2, **P < 0.01 vs Sham, ##P < 0.01 vs +shNC; n = 5. (C) qRT-PCR analysis of the CACNA1C mRNA level; **P < 0.01 vs sham; #P < 0.05 vs +shNC; n = 4. (D–F) Representative traces of the whole-cell ICaL and I-V relationships of the ICaL, n = 6 cells. Data are presented as the mean ± SEM.

In the present study, we investigated the potential role of CDR1as in regulating cardiac calcium channels following MI. We used an adeno-associated virus (AAV9) to deliver shCDR1as and shNC into mice via tail vein injection and validated their knockdown efficiency ( Supplementary Figure S1A,B), establishing an MI model after a four-week period ( Figure 1A). The successful construction of the MI mouse model was initially verified via electrocardiogram (ECG) and TTC staining. Concurrently, ST-segment elevation accompanied by arrhythmia was detected in the electrocardiograms of the MI mice ( Supplementary Figure S2). Our findings revealed that the knockdown of *CDR1as* can prevent the decrease in Cav1.2 expression that occurs after MI ( Figure 1B,C). Consistent with the reduction in Cav1.2 levels observed in MI mice, the downregulation of CDR1as post-MI led to an improvement in the reduction in *I*_CaL_ ( Figure 1D–F). Overexpression of CDR1as further diminishes *I*_CaL_ in mice with MI ( Supplementary Figure S3).

### Overexpression of CDR1as causes defects in the Cav1.2 channel

**Figure 2:**
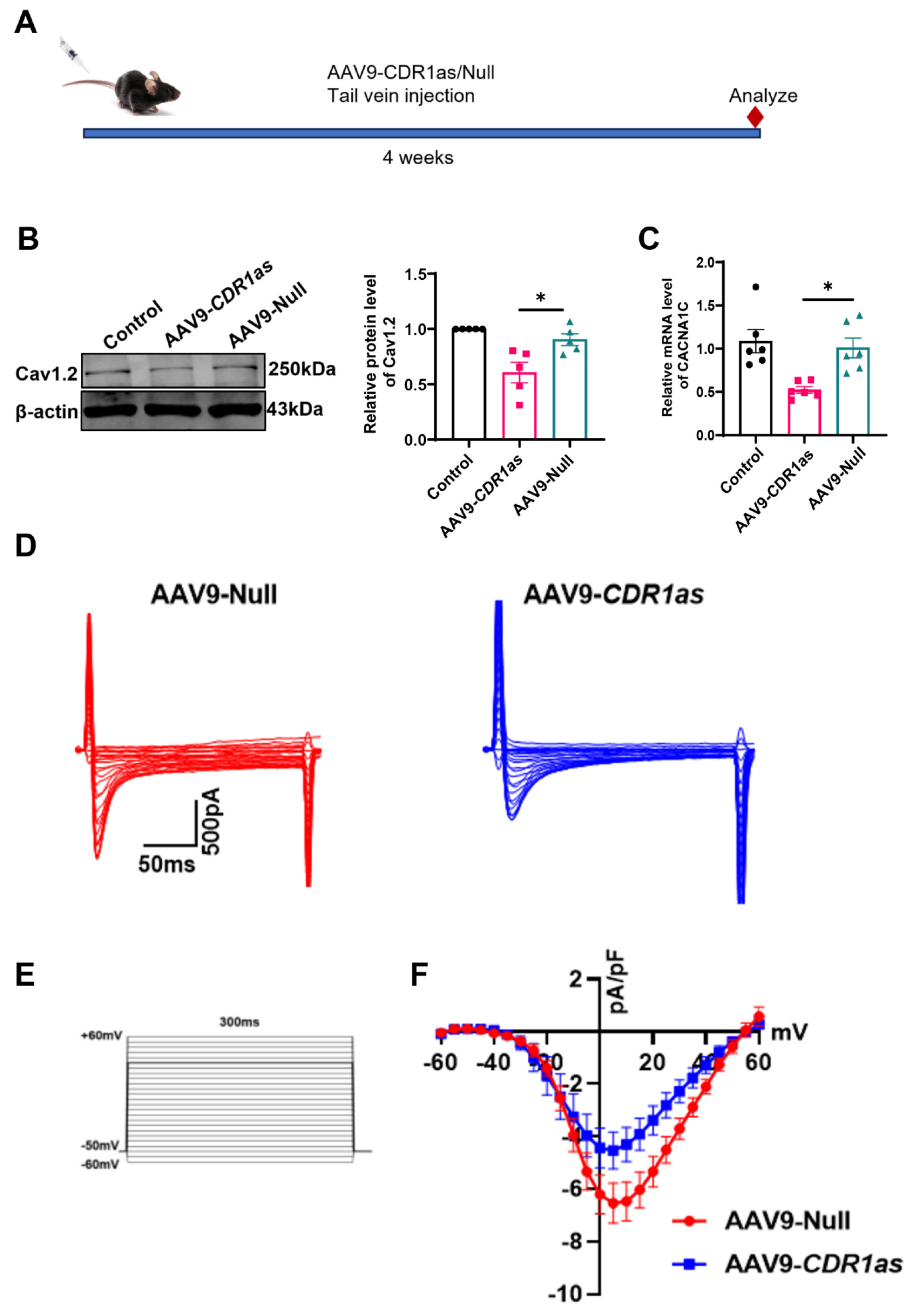
Figure 2 CDR1as can decrease Cav1.2 expression and *I*_CaL_ in ventricular myocytes (A) Experimental procedures for AAV9 injection and examinations (AAV9-CDR1as, AAV9-Null). (B) Western blot analysis of Cav1.2 protein expression in mice four weeks after tail vein injection of AAV9-CDR1as or AAV9-Null, *P < 0.05 vs AAV9-Null; n = 5. (C) qRT-PCR analysis of CACNA1C mRNA levels in mice four weeks after tail vein injection of AAV9-CDR1as or AAV9-Null, *P < 0.05 vs AAV9-Null; n = 6. (D–F) Representative traces of the whole-cell ICaL and I-V relationships of the ICaL, n = 6 cells. Data are presented as the mean ± SEM.

To further investigate the role of CDR1as in the heart, we implemented a gain-of-function strategy for CDR1as overexpression in mouse cardiomyocytes by administering AAV9-CDR1as ( Figure 2A) and verified the overexpression efficiency ( Supplementary Figure S1C). We evaluated the change in the Cav1.2 channel after CDR1as overexpression. The expression of Cav1.2 in heart tissue was slightly lower in mice treated with AAV9-CDR1as than in those treated with AAV9-Null ( Figure 2B,C). Consistently, we observed that overexpression of CDR1as led to a significant reduction in the density of the *I*_CaL_ in isolated ventricular myocytes, as depicted in Figure 2D–F.

### *CDR1as* knockdown improves Cav1.2 channel expression in hypoxic cardiomyocytes

**Figure 3:**
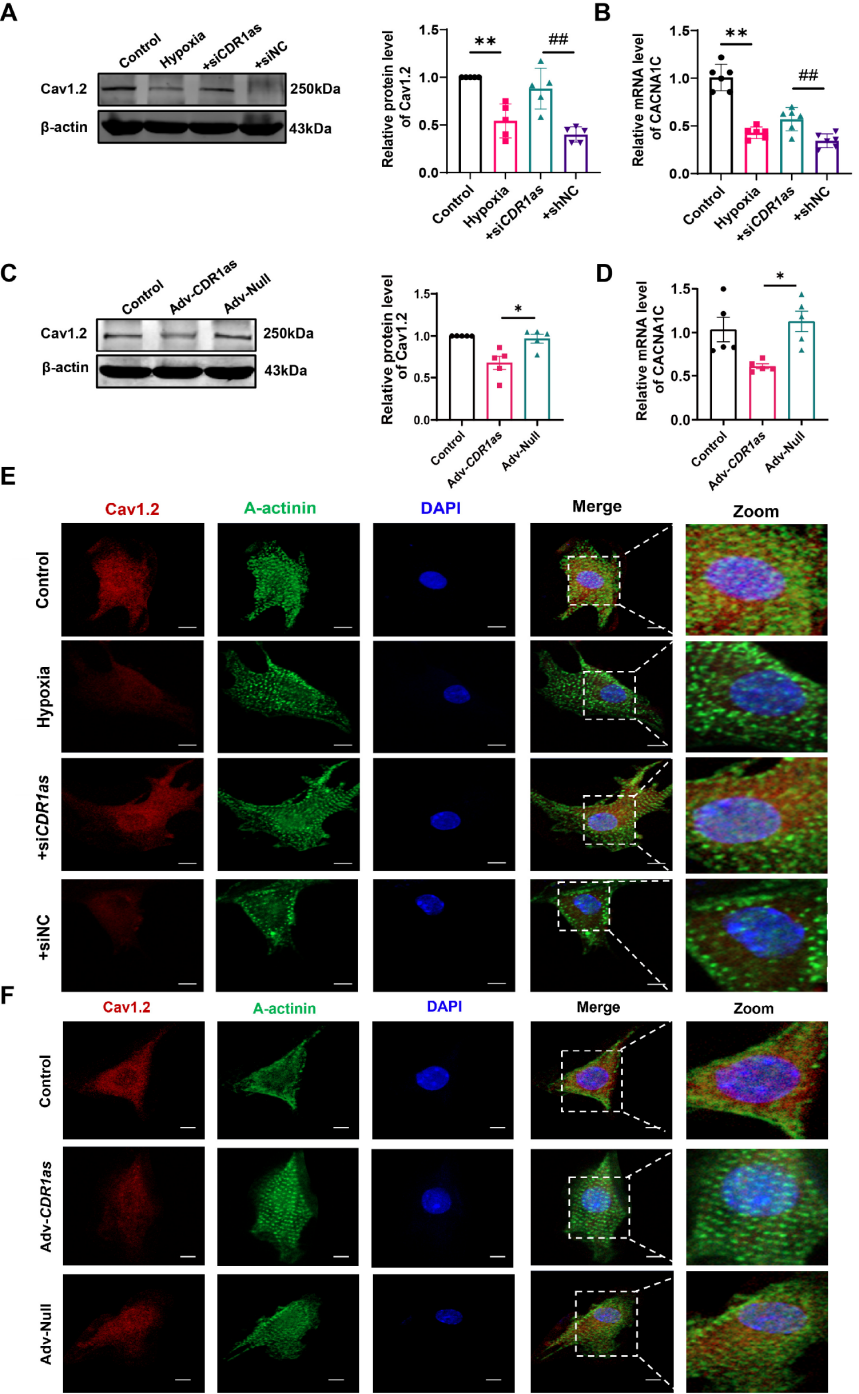
Figure 3 CDR1as downregulates the expression of Cav1.2 at the protein and mRNA levels (A) Representative bands and quantitative analysis of Cav1.2 in hypoxic NMCMs. **P < 0.01 vs Control, ##P < 0.01 vs +siNC; n = 5. (B) mRNA expression of CACNA1C in hypoxic NMCMs. **P < 0.01 vs Control, ##P < 0.01 vs +siNC; n = 6. (C) Representative bands and quantitative analysis of Cav1.2 in NMCMs after treatment with Adv-CDR1as and Adv-Null. *P < 0.05 vs Adv-Null; n = 5. (D) CACNA1C mRNA expression in NMCMs 48 h after infection with Adv-CDR1as or Adv-Null. *P < 0.05 vs Adv-Null; n = 5. (E,F) Immunofluorescence assay visualizing the expression levels of Cav1.2 proteins in NMCMs, n = 6. Scale bar: 20 μm. Data are presented as the mean ± SEM.

Next, we further verified the influence of CDR1as on calcium channels through *in vitro* experiments. The siRNA containing the *CDR1as* gene was transfected into neonatal mouse cardiomyocytes (NMCMs) for 48 h, followed by 12 h of hypoxia treatment. Firstly, the successful establishment of the *in vitro* cardiomyocyte hypoxia model was confirmed via CCK8 ( Supplementary Figure S4A) and LDH release assays ( Supplementary Figure S4B). Subsequently, silencing of *CDR1as* significantly reversed the hypoxia-induced reduction in Cav1.2 expression ( Figure 3A,B). Afterwards, adenovirus-mediated overexpression of CDR1as (Adv-CDR1as) was introduced into NMCMs for a duration of 48 h. The results revealed that the overexpression of CDR1as resulted in a decrease in cell viability ( Supplementary Figure S4C) and an increase in LDH release ( Supplementary Figure S4D). In agreement with the results obtained from the knockdown experiments, the forced expression of CDR1as was accompanied by a notable downregulation of Cav1.2 channel expression, as illustrated in ( Figure 3C,D). In accordance with the expression level of the Cav1.2 protein, the fluorescence intensity of Cav1.2 was also diminished in the presence of elevated CDR1as levels, as depicted in ( Figure 3E,F).

### Knockdown of *CDR1as* improves cardiac contractile function in MI model mice

**Figure 4:**
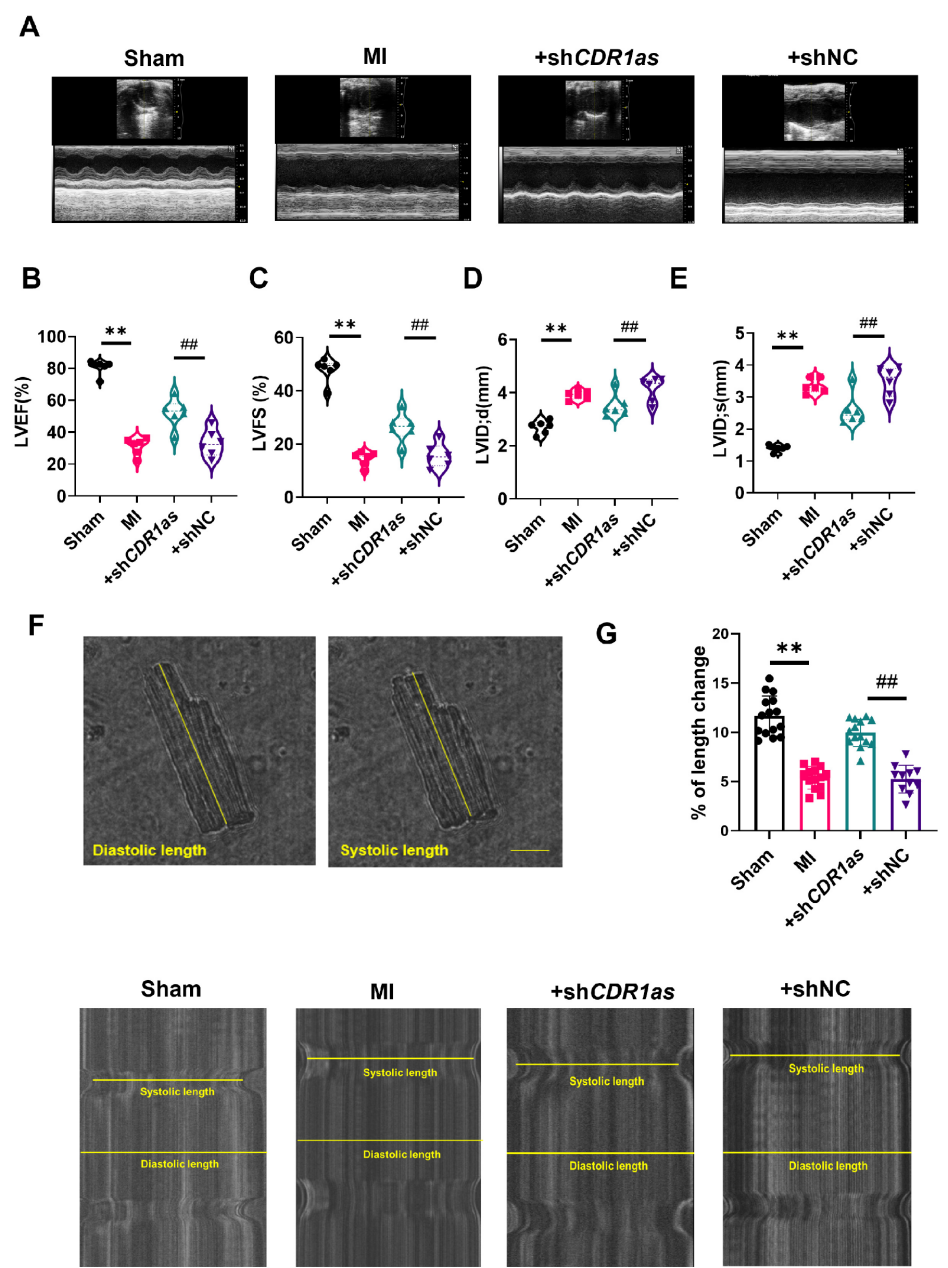
Figure 4 Knockdown of *CDR1as* can improve cardiac function after MI in mice (A) Representative short-axis M-mode echocardiographs of the sham group, MI group, MI +shCDR1as group and MI +shNC group. (B–E) Statistics of left ventricular ejection fractions (LVEF), left ventricular fractional shortening (LVFS), enlargement of the left ventricular internal dimension at end-diastole (LVIDd) and the left ventricular internal dimension at systole (LVIDs). **P < 0.01 vs Sham, ##P < 0.01 vs +shNC; n = 6. (F) Changes in the systolic length and diastolic length of isolated single ventricular myocytes. Scale bar: 20 μm. (G) Effect of CDR1as knockdown on sarcomere shortening (SS). **P < 0.01 vs Sham, ##P < 0.01 vs +shNC; n = 11–15. Data are presented as the mean ± SEM.

Calcium, which serves as a crucial second messenger, is pivotal in electrophysiology, excitation-contraction coupling processes and muscular contraction [ 20, 21]. Cav1.2 is the predominant subtype of L-type calcium channel in the brain, heart and smooth muscle. This subtype is indispensable for the proper contraction of both cardiac and smooth muscle, thereby playing a fundamental role in maintaining the physiological function and integrity of these tissues [22]. Given the effects of Cav1.2 mentioned above, we next examined the influence of CDR1as on cardiomyocyte contractile function. We first employed echocardiography to investigate the function of CDR1as in cardiac systolic function *in vivo*. As depicted in ( Figure 4A–E), the left ventricular ejection fraction (LVEF) and left ventricular short-axis shortening rate (LVFS) were markedly lower in the MI group than in the sham group. Concurrently, both the left ventricular end-diastolic diameter (LVIDd) and the left ventricular end-systolic diameter (LVIDs) increased. Notably, these cardiac function parameters were mitigated significantly by the knockdown of *CDR1as*, suggesting a potential role for CDR1as in modulating cardiac function following MI.

The consequences related to the impact of CDR1as on cardiac systolic function, as elucidated by the preceding findings, suggest that CDR1as has a deleterious effect on the mechanical performance of the heart. To corroborate this inference, we isolated ventricular myocytes from MI mice and *CDR1as*-knockdown mice and measured cell shortening to assess myocardial contractility. As depicted in ( Figure 4F,G), a marked reduction in the shortening of myocardial cells was observed in the MI group compared with the sham group, indicating the impairment of cardiac contractility resulted from MI. Nevertheless, the knockdown of *CDR1as* led to a significant improvement in myocyte shortening, mitigating the effects of MI. Overexpression of CDR1as further reduced the cardiac contractile function of MI mice ( Supplementary Figure S5). In summary, our data suggest that knockdown of *CDR1as* improves cardiac systolic function in mice with MI, shedding light on a potential therapeutic target for ameliorating cardiac function post-MI.

### CDR1as has no significant effect on atrial electrophysiology

**Figure 5:**
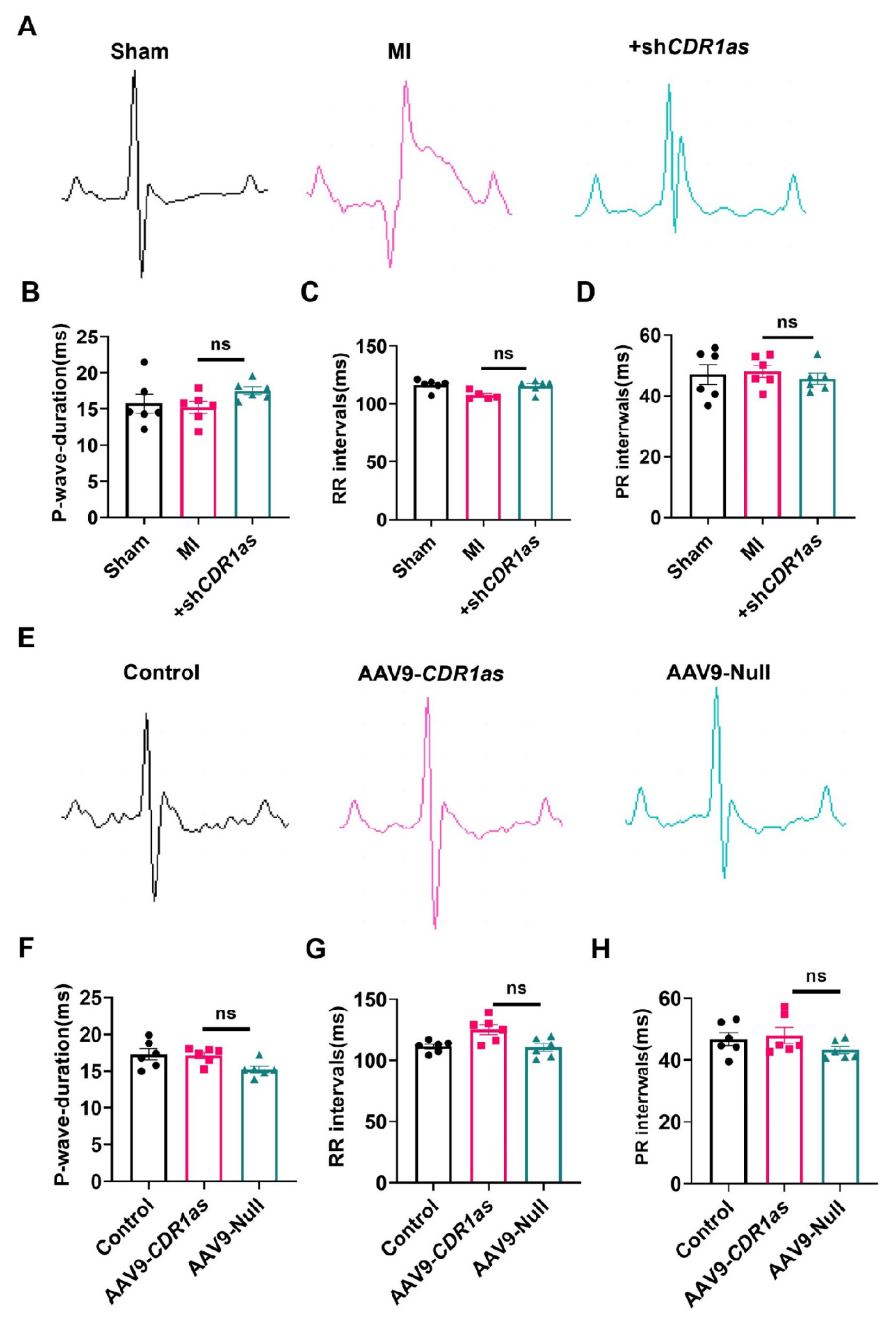
Figure 5 CDR1as has no effect on atrial arrhythmias (A,E) Examples of surface ECGs. (B–D,F–H) CDR1as had no effect on atrial arrhythmia indices such as (B,F) P wave duration, (C,G) RR interval, and (D,H) PR interval. n = 6. Data are presented as the mean ± SEM.

Our previous investigations confirmed that the QRS wave group and QT/QTc interval are prolonged in mice after MI [10]. To further clarify that CDR1as exerts its effect by affecting ventricular electrophysiology rather than atrial electrophysiology, we proceeded to verify our idea via ECG analysis. The ECG recordings revealed no statistically significant differences in the RR interval, P-wave duration or PR interval between *CDR1as*-knockdown mice and MI mice ( Figure 5A–D), suggesting that atrial electrophysiology remained unaffected. The ECG records of the mice overexpressing CDR1as further support the aforementioned conclusions ( Figure 5E–H). This comprehensive assessment of the ECG parameters not only bolsters our understanding of the specific electrophysiological mechanisms involved but also highlights the potential implications of CDR1as regarding cardiac function and its disorders.

## Discussion

VAs constitute a primary factor contributing to mortality among patients with MI, substantially augmenting the likelihood of sudden cardiac death [23]. Despite improvements in treatments, survivors of MI remain at an elevated risk of developing VAs and sudden cardiac death [24], which underscores the critical need for further research to mitigate the potentially fatal consequences associated with post-MI VAs. In this study, we demonstrated that CDR1as is involved in post-infarction arrhythmias by altering calcium processing in cardiomyocytes. Notably, we initially reported that the downregulation of CDR1as mitigates ischemia-induced Cav1.2 dysfunction, thereby reducing the incidence of VAs after MI. This research highlights that targeting CDR1as through a novel potential mechanism could hold promise in the treatment of ischemic arrhythmias. Further bioinformatics analysis showed that the CDR1as is highly conserved across species as indicated in Supplementary Figure S6.

Non-coding RNAs, encompassing miRNAs, lncRNAs and circRNAs, have been recognized for their pivotal roles in the genesis and progression of VAs [25]. A previous study revealed that lncRNA-TCONS-00075467 can function as a molecular sponge for miR-328, targeting the L-type calcium channel gene *CACNA1C*, which is integral to atrial electrical remodeling [26]. Furthermore, lncRNA-MALAT1 contributes to arrhythmogenesis by inhibiting the myocardial transient outward potassium current ( *I*_to_) [27]. This inhibitory effect on *I*_to_ disrupts normal electrical activity within the myocardium, leading to increased susceptibility to arrhythmic events. Our group’s previous research established the impact of circRNA CDR1as on sodium (Na ^+^) and potassium (K ^+^) channels following MI. Nevertheless, the function of CDR1as in calcium (Ca ^2+^) channel regulation and its broader implications for arrhythmia management have yet to be fully understood and clarified [ 10, 28]. Hence, a comprehensive and profound exploration of the role of non-coding RNA in arrhythmias is of paramount clinical significance, offering valuable insights for the prevention and management of cardiovascular diseases.

The remodeling of ion channels, disruption of calcium homeostasis, and increased myofilament sensitivity to Ca ^2+^ may underlie the alterations in APD, formation of reentry circuits, and initiation of triggered activity. These events, including early after depolarizations (EADs) and delayed after depolarizations (DADs), are critical in the genesis of VAs observed in hypertrophic cardiomyopathy (HCM) [29]. Defects in intracellular Ca ^2+^ homeostasis have been identified as crucial determinants of the development of arrhythmias in acquired heart disease [ 14– 30]. EADs and DADs have been implicated in the induction of arrhythmias across a diverse range of heart diseases [31]. The alteration of APD is a principal contributor to the development of arrhythmia. Action potential prolongation provides a substrate for EADs, which in turn serve as triggers of cardiac arrhythmias [32]. Our previous studies revealed that knockdown of *CDR1as* can shorten the APD in MI model mice [10]. The opening of the *I*_CaL_, which initiates the internal flow of calcium ions, is essential for determining the length of the platform and the APD. After MI, inactivation of *I*_CaL_ channels is postponed, leading to early post-depolarization and increasing the risk of arrhythmia [33]. In general, EADs and DADs coexist and influence each other. This is because the membrane voltage is strongly influenced by the current of calcium-sensitive ions, and Ca-voltage coupling can facilitate complex AP dynamics [34].

*I*_CaL_ plays a prominent role in the formation and propagation of EAD [35]. A reduction in the LTCC current could mitigate Ca ^2+^ influx, thereby protecting the heart during stress stimuli that precipitate disease [36]. Nevertheless, Fu and colleagues reported that 7 days post-MI in mice, there was a delay in the inactivation of *I*_CaL_ channels. This delay results in early post depolarizations, creating a substrate prone to arrhythmia [4]. Furthermore, the absence of Mettl3 leads to an aberrant decrease in Cav1.2 protein expression and *I*_CaL_, thereby increasing susceptibility to VAs [37]. Interestingly, our observations revealed an unexpected phenomenon: reduced expression levels of Cav1.2 and *I*_CaL_ were found to be instrumental in the genesis of VAs after MI. Moreover, we demonstrated that this propensity could be significantly ameliorated by the targeted suppression of CDR1as. This finding aligns well with previously reported alterations in Cav1.2 expression within ventricular myocytes [ 37, 38]. This correlation not only reinforces the importance of Cav1.2 in cardiac electrophysiology but also suggests for the first time that a reduction in LTCC activity in cardiomyocytes may increase the incidence of arrhythmias after MI.

Furthermore, our research revealed that knockdown of the *CDR1as* gene exerts a protective effect on cardiac function in mice with MI. This protective effect is achieved through the upregulation of Cav1.2 expression, which in turn plays a crucial role in maintaining the normal physiological function of the heart. The disparities noted in Cav1.2 expression could stem from variations in experimental models or the time points at which the experiments are conducted. Within the initial hour following an MI, the likelihood of VAs can increase to 10%–40% [39]. Consequently, it is logical to deduce that within 12 h post-MI, the body has yet to transition into a compensatory phase. During this interval, the diminished expression of Cav1.2 is likely to impair cardiac function, thereby predisposing the individual to the onset of VAs. The reduction of ion channels is intrinsically arrhythmia-inducing, which is consistent with the genetic syndrome of reduced ion channels. Overall, these data suggest that MI can alter cardiac calcium expression and increase the vulnerability of mice to VAs after MI.

A multitude of proteins pivotal to the calcium cycle, such as sarcoplasmic reticulum calcium ATPase 2a (SERCA2a) and ryanodine receptor 2 (RyR2), work in concert to regulate the transport and circulation of calcium within the cell, thereby maintaining intracellular calcium homeostasis [ 20, 40, 41]. Nevertheless, CDR1as has no significant effect on the RyR2 protein, as shown in Supplementary Figure S7A,B. Although CDR1as plays a role in regulating SERCA2a expression ( Supplementary Figure S7C,D), the calmodulin associated with calcium currents is predominantly Cav1.2. Therefore, this study focuses primarily on the influence of CDR1as on Cav1.2. Our study, while illuminating, is not without its limitations. At present, we hypothesize that CDR1as may regulate the expression of Cav1.2 by modulating Cav1.2 mRNA methylation. In addition, circular RNAs (circRNAs) can also bind to RNA-binding proteins (RBPs) and regulate the activity of RBPs, thereby affecting signal transduction and gene expression regulation [42]. Furthermore, we hypothesized that CDR1as may be an endogenous inhibitor of Cav1.2. By binding to the Cav1.2 protein, CDR1as may limit the expression level of Cav1.2 within cells and thus inhibit its activity. Our follow-up studies will be devoted to uncovering this mystery.

In conclusion, our investigation reveals an unprecedented role for CDR1as in arrhythmias occurring after MI. We demonstrate that CDR1as triggers a reduction in Cav1.2, which subsequently results in a decrease in the calcium current and subsequent electrophysiological disruptions. Furthermore, this disruption of calcium homeostasis results in abnormal cardiac systolic function, further increasing the risk of arrhythmia. This does not conflict with the previous involvement of CDR1as in arrhythmogenesis through Nav1.5 and Kir6.2 but rather emphasizes the complexity and multiplicity of the roles of CDR1as in arrhythmias after MI, suggesting that different channels may be involved in different aspects or subsets of the arrhythmia phenotype. This research opens new avenues in the quest for treatments targeting arrhythmias post-MI, offering a promising direction for future therapeutic strategies.

In addition, circRNA applications have focused on protein expression in gene therapy, CAR-T-cell therapy, protein replacement and vaccines, and approaches of clinical translational value have received much attention [17]. Based on the conclusions reached in this work, it is necessary to consider exosome-encapsulated shCDR1as as a possible clinical therapy for the treatment of MI or ischemic arrhythmia. Moreover, virus-like particles (VLPs), which are spontaneously assembled from viral structural proteins that are effective in tissue targeting and delivery, may also constitute our first choice [ 43, 44]. Consequently, we hypothesize that VLPs can be harnessed to engineer and fabricate prophylactic vaccines against CDR1as that specifically target cardiomyocytes, thereby showing great promise for further development.

## Supplementary Data

Supplementary data is available at *Acta Biochimica et Biophysica Sinica* online.

## Supporting information

24840-Supplementary_materia_C2

## References

[1] Fu D (2015). Cardiac arrhythmias: diagnosis, aymptoms, and treatments. Cell Biochem Biophys.

[2] Ray L, Geier C, DeWitt KM (2023). Pathophysiology and treatment of adults with arrhythmias in the emergency department, part 2: ventricular and bradyarrhythmias. Am J Health- Syst Pharm.

[3] Hammerer-Lercher A, Namdar M, Vuilleumier N (2020). Emerging biomarkers for cardiac arrhythmias. Clin Biochem.

[4] Fu H, Shuai W, Kong B, Jiang X, Huang H (2021). MD1 Depletion Predisposes to Ventricular Arrhythmias in the Setting of Myocardial Infarction. Heart Lung Circ.

[5] Bonny A, Ngantcha M, Scholtz W, Chin A, Nel G, Anzouan-Kacou JB, Karaye KM (2019). Cardiac arrhythmias in africa. J Am Coll Cardiol.

[6] Huang Q, Chu Z, Wang Z, Li Q, Meng S, Lu Y, Ma K (2024). circCDK13-loaded small extracellular vesicles accelerate healing in preclinical diabetic wound models. Nat Commun.

[7] Dinh PS, Tran CMT, Dinh TPH, Ali A, Pan SL (2024). Hsa_circRNA_0000284 acts as a ceRNA to participate in coronary heart disease progression by sponging miRNA-338-3p via regulating the expression of
*ETS1*. J Biomol Structure Dyn.

[8] Yin L, Li L, Gao M, Qi Y, Xu L, Peng J (2024). circMIRIAF aggravates myocardial ischemia-reperfusion injury via targeting miR-544/WDR12 axis. Redox Biol.

[9] Knierim M, Bommer T, Paulus M, Riedl D, Fink S, Pöppl A, Reetz F (2024). Cellular calcium handling and electrophysiology are modulated by chronic physiological pacing in human induced pluripotent stem cell-derived cardiomyocytes. Am J Physiol-Heart Circulatory Physiol.

[10] Liu Y, Wang J, Zhao X, Li W, Liu Y, Li X, Zhao D (2023). CDR1as promotes arrhythmias in myocardial infarction via targeting the NAMPT-NAD+ pathway. Biomed Pharmacother.

[11] Tallo FS, de Santana PO, Pinto SAG, Lima RY, de Araújo EA, Tavares JGP, Pires-Oliveira M (2023). Pharmacological modulation of the Ca2+/cAMP/Adenosine signaling in cardiac cells as a new cardioprotective strategy to reduce severe arrhythmias in myocardial infarction. Pharmaceuticals.

[12] Hamilton S, Terentyev D (2022). ER stress and calcium-dependent arrhythmias. Front Physiol.

[13] Vandewiele F, Pironet A, Jacobs G, Kecskés M, Wegener J, Kerselaers S, Hendrikx L (2022). TRPM4 inhibition by meclofenamate suppresses Ca2+-dependent triggered arrhythmias. Eur Heart J.

[14] Hamilton S, Veress R, Belevych A, Terentyev D (2021). The role of calcium homeostasis remodeling in inherited cardiac arrhythmia syndromes. Pflugers Arch.

[15] Ho-Xuan H, Glažar P, Latini C, Heizler K, Haase J, Hett R, Anders M (2020). Comprehensive analysis of translation from overexpressed circular RNAs reveals pervasive translation from linear transcripts. Nucleic Acids Res.

[16] Jiang S, Jiao G, Chen Y, Han M, Wang X, Liu W (2020). AstragalosideIV attenuates chronic intermittent hypoxia-induced myocardial injury by modulating Ca
^2+^ homeostasis. Cell Biochem Function.

[17] Wang D, Tai PWL, Gao G (2019). Adeno-associated virus vector as a platform for gene therapy delivery. Nat Rev Drug Discov.

[18] Zogg H, Singh R, Ro S (2022). Current advances in RNA therapeutics for human diseases. Int J Mol Sci.

[19] Nassal DM, Hund TJ (2022). Peering into the molecular machinery for regulation of Cav1.2 channel clusters. Circ Res.

[20] Imoto K, Hirakawa M, Okada M, Yamawaki H (2018). Canstatin modulates L-type calcium channel activity in rat ventricular cardiomyocytes. Biochem Biophys Res Commun.

[21] Hidalgo C (2017). Calcium Rules. Circulation.

[22] Gao S, Yao X, Chen J, Huang G, Fan X, Xue L, Li Z (2023). Structural basis for human Cav1.2 inhibition by multiple drugs and the neurotoxin calciseptine. Cell.

[23] Li J, Xu C, Liu Y, Li Y, Du S, Zhang R, Sun Y (2020). Fibroblast growth factor 21 inhibited ischemic arrhythmias via targeting miR-143/EGR1 axis. Basic Res Cardiol.

[24] Gardner RT, Wang L, Lang BT, Cregg JM, Dunbar CL, Woodward WR, Silver J (2015). Targeting protein tyrosine phosphatase σ after myocardial infarction restores cardiac sympathetic innervation and prevents arrhythmias. Nat Commun.

[25] Prestes PR, Maier MC, Woods BA, Charchar FJ (2020). A guide to the short, long and circular RNAs in hypertension and cardiovascular disease. Int J Mol Sci.

[26] Li Z, Wang X, Wang W, Du J, Wei J, Zhang Y, Wang J (2017). Altered long non-coding RNA expression profile in rabbit atria with atrial fibrillation: TCONS_00075467 modulates atrial electrical remodeling by sponging miR-328 to regulate CACNA1C. J Mol Cell Cardiol.

[27] Zhu P, Yang M, Ren H, Shen G, Chen J, Zhang J, Liu J (2018). Long noncoding RNA MALAT1 downregulates cardiac transient outward potassium current by regulating miR-200c/HMGB1 pathway. J Cell Biochem.

[28] van Opbergen CJM, den Braven L, Delmar M, van Veen TAB (2019). Mitochondrial dysfunction as substrate for arrhythmogenic cardiomyopathy: a search for new disease mechanisms. Front Physiol.

[29] Shen H, Dong SY, Ren MS, Wang R (2022). Ventricular arrhythmia and sudden cardiac death in hypertrophic cardiomyopathy: from bench to bedside. Front Cardiovasc Med.

[30] Penitente AR, Novaes RD, Silva ME, Silva MF, Quintão-Júnior JF, Guatimosim S, Cruz JS (2014). Basal and β-adrenergic cardiomyocytes contractility dysfunction induced by dietary protein restriction is associated with downregulation of SERCA2a expression and disturbance of endoplasmic reticulum Ca
^2+^ regulation in rats. Cell Physiol Biochem.

[31] Song Z, Ko CY, Nivala M, Weiss JN, Qu Z (2015). Calcium-voltage coupling in the genesis of early and delayed afterdepolarizations in cardiac myocytes. Biophys J.

[32] Li D, Xue G, Yang J, Li C, Zhang R, Tian T, Li Z (2022). Knockout of interleukin-17A diminishes ventricular arrhythmia susceptibility in diabetic mice via inhibiting NF-κB-mediated electrical remodeling. Acta Pharmacol Sin.

[33] Fo Y, Zhang C, Chen X, Liu X, Ye T, Guo Y, Qu C (2020). Chronic sigma-1 receptor activation ameliorates ventricular remodeling and decreases susceptibility to ventricular arrhythmias after myocardial infarction in rats. Eur J Pharmacol.

[34] Qu Z, Hu G, Garfinkel A, Weiss JN (2014). Nonlinear and stochastic dynamics in the heart. Phys Rep.

[35] Xie LH, Chen F, Karagueuzian HS, Weiss JN (2009). Oxidative stress–induced afterdepolarizations and calmodulin kinase II signaling. Circ Res.

[36] Liu M, Liu H, Parthiban P, Kang GJ, Shi G, Feng F, Zhou A (2021). Inhibition of the unfolded protein response reduces arrhythmia risk after myocardial infarction. J Clin Invest.

[37] Shi L, Jin X, Li Z, Gong R, Guo Y, Ma J, Zhang Y (2022). Mettl3 deficiency leads to the upregulation of Cav1.2 and increases arrhythmia susceptibility in mice. Acta Biochim Biophys Sin.

[38] Cui G, Xin Q, Tseng HHL, Hoi MPM, Wang Y, Yang B, Choi IL (2018). A novel Ca2+ current blocker promotes angiogenesis and cardiac healing after experimental myocardial infarction in mice. Pharmacol Res.

[39] Frampton J, Ortengren AR, Zeitler EP (2023). Arrhythmias after acute myocardial infarction. Yale J Biol Med.

[40] Stammers AN, Susser SE, Hamm NC, Hlynsky MW, Kimber DE, Kehler DS, Duhamel TA (2015). The regulation of sarco(endo)plasmic reticulum calcium-ATPases (SERCA). Can J Physiol Pharmacol.

[41] Jiao L, Li M, Shao Y, Zhang Y, Gong M, Yang X, Wang Y (2019). lncRNA-ZFAS1 induces mitochondria-mediated apoptosis by causing cytosolic Ca2+ overload in myocardial infarction mice model. Cell Death Dis.

[42] Li H, Yang F, Hu A, Wang X, Fang E, Chen Y, Li D (2019). Therapeutic targeting of
*circ-CUX 1*/EWSR 1/MAZ axis inhibits glycolysis and neuroblastoma progression. EMBO Mol Med.

[43] Kheirvari M, Liu H, Tumban E (2023). Virus-like particle vaccines and platforms for vaccine development. Viruses.

[44] Fuenmayor J, Gòdia F, Cervera L (2017). Production of virus-like particles for vaccines. New Biotechnol.

