# Supplementary material for: CDR1as modulates arrhythmia post-myocardial infarction via regulating Cav1.2: CDR1as damages the Cav1.2 channel in the heart
Source: Acta Biochim Biophys Sin (Shanghai). 2025 Jul 29;57(11):1780–90. doi: 10.3724/abbs.2025126 (PMC12666664; doi:10.3724/abbs.2025126)
Supplement: 24840-Supplementary_materia_C2 [file 24840-Supplementary_materia_C2.docx]

**CDR1as Modulates Arrhythmia Post-Myocardial Infarction via Regulating Cav1.2**

Jiapan Wang^1^, Wenjie Liao^1^, Xingda Li^1^, Zhen Chen^1^, Chunlei Duan^1^, Zhenru Wang^1^, Hongda Li^1^, Haonan Du^1^, Ye Yuan^1^, Zhimin Du^1,2^ *

^1^Institute of Clinical Pharmacology, National Key Laboratory of Frigid Cardiovascular Disease, the Second Affiliated Hospital of Harbin Medical University, Harbin, 150001, China; ^2^State Key Laboratory of Quality Research in Chinese Medicines, Macau University of Science and Technology, Macau, 999078, China

*Correspondence address. Tel: +86-86605353; E-mail: [dzm1956@126.com](mailto:dzm1956@126.com)

**Supplementary Table 1.** The sequence of the siRNAs

| Gene name | Sense (5'→ 3') Antisense (5'→3') | |
| --- | --- | --- |
| siCDR1as | GUCCAAGUCUUCCCAGAAATT | UUUCUGGGAAGACUUGGACTT |
| siNC | UUCUCCGAACGUGUCACGUTT | ACGUGACACGUUCGGAGAATT |

**Supplementary Table 2.** The primers used for real-time PCR

| Gene name | Primer sequence (5'→3') |
| --- | --- |
| Mouse CDR1as-F | TCTGCTCGTCTTCCAACATC |
| Mouse CDR1as-R | AGATCAGCAC ACTGGAGAC |
| β-actin-F | ACTGCCGAATCCTCTTCCT |
| β-actin-R | TCAACGTCACACTTCATGATGGA |
| CACNA1C-F | CCTGGAACGAGTGGAGTATC |
| CACNA1C-R | CATTGCGGAGGTAAGCGTTG |

**Supplementary Figures and Figure legends**

**
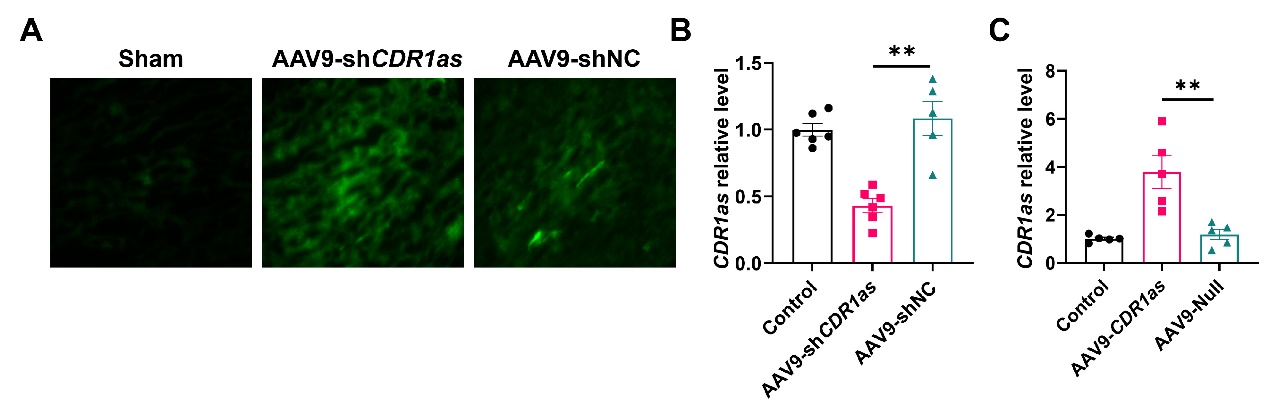
**

**Fig****ure S1. Verification of the efficiency of the circRNA CDR1as vector** (A) Representative cardiac sections showing the successful delivery of AAV9-shCDR1as into heart tissue. (B) Verification of the efficiency of AAV9-shCDR1as in knocking down endogenous CDR1as in mouse myocardial tissues, as determined by qRT-PCR. ^**^*P* < 0.01 *vs*. AAV9-shNC; *n* = 5‒6. (C) Verification of the overexpression of CDR1as elicited by AAV9-CDR1as in normal mice. ^**^*P* < 0.01 *vs*. AAV9-Null; *n* = 5. The data are presented as the mean ± SEM.


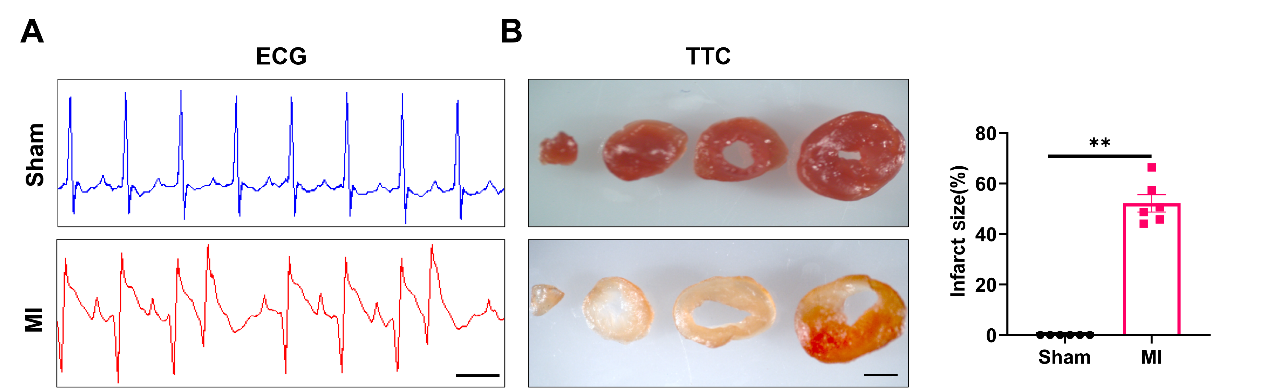


**Figure S2. A mouse model of MI was successfully established** (A) Representative ECG tracings of each group. *n*=8. Scale bars: 0.1 s. (B) Representative photographs and quantiﬁcation of the infarct area as a percentage of the total left ventricular (LV) area by TTC staining, ^**^*P* < 0.01 *vs*. the sham group; *n*=6. The infarct areas are presented in white, whereas the normal areas are red. Scale bars: 2 mm.

**
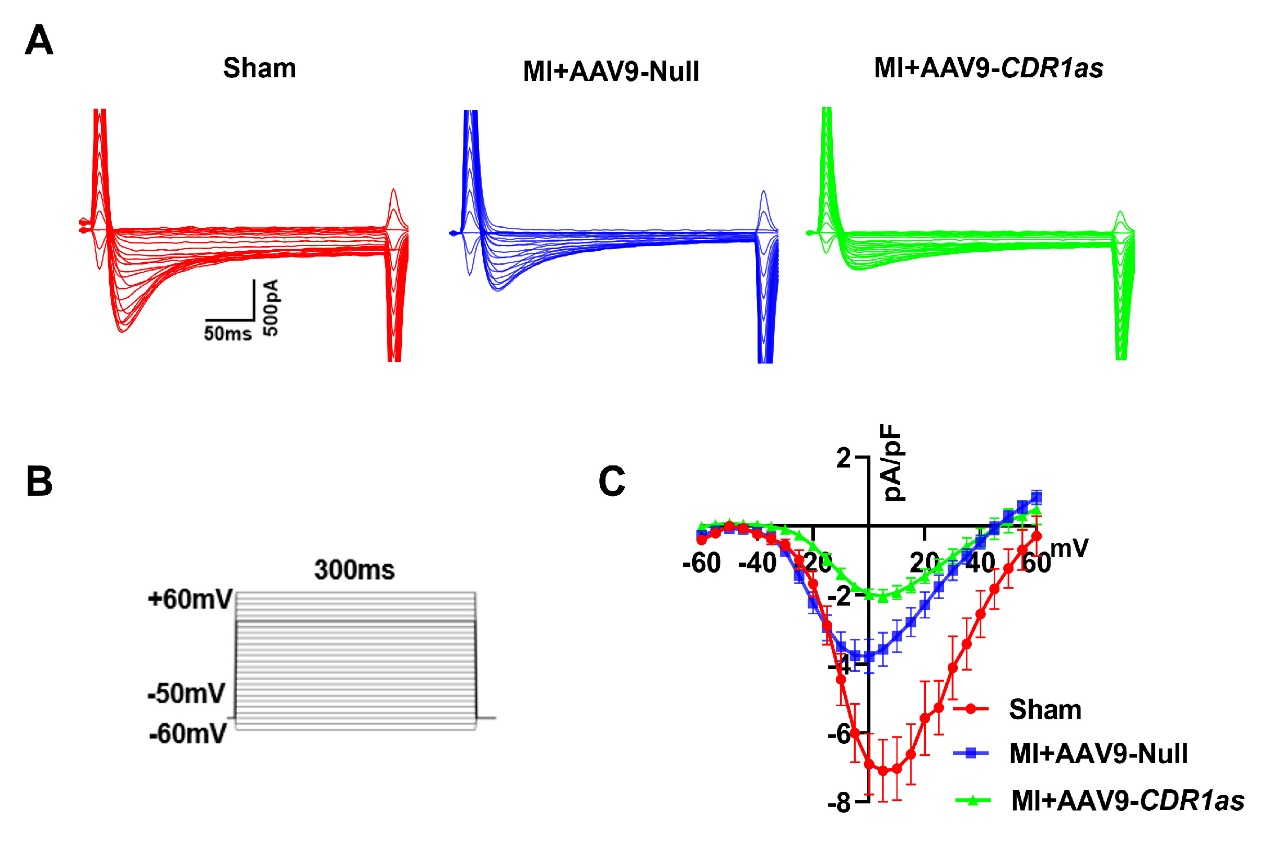
**

**Figure S3. CDR1as overexpression reduces *I*_CaL_ in MI mice** Representative traces of the whole-cell *I*_CaL_ and I-V relationships of *I*_CaL_, ^*^*P* < 0.05 *vs.* MI+AAV9-Null; *n*=6 cells. The data are presented as the mean ± SEM.

**
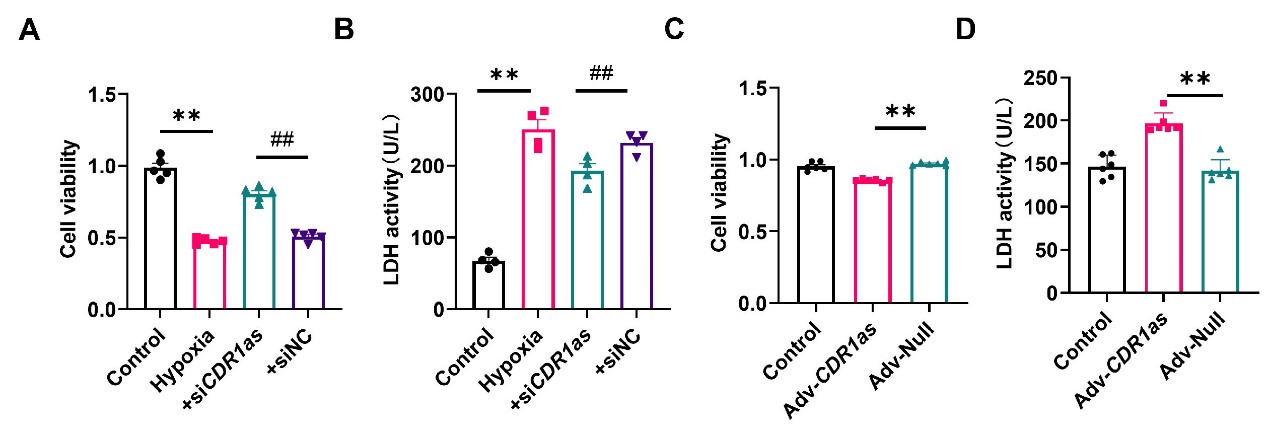
**

**Figure S4. Knockdown of CDR1as inhibits hypoxia-induced cardiomyocyte injury** (A) Changes in the viability of cardiomyocytes after the knockdown of CDR1as. ^**^*P* < 0.01 *vs.* Control, ^##^ *P* < 0.01 *vs*. siNC; *n*=5. (B) Changes in LDH levels released from cardiomyocytes after the knockdown of CDR1as. ^**^*P* < 0.01 *vs.* Control, ^##^*P* < 0.01 *vs*. siNC; *n*=4. (C) Changes in the viability of cardiomyocytes after the overexpression of CDR1as. ^**^*P* < 0.01 *vs.* Adv-Null; *n*=7. (D) Changes in LDH content released from cardiomyocytes after the overexpression of CDR1as. ^**^*P* < 0.01 *vs.* Adv-Null; *n*=6. The data are presented as the mean ± SEM.

**
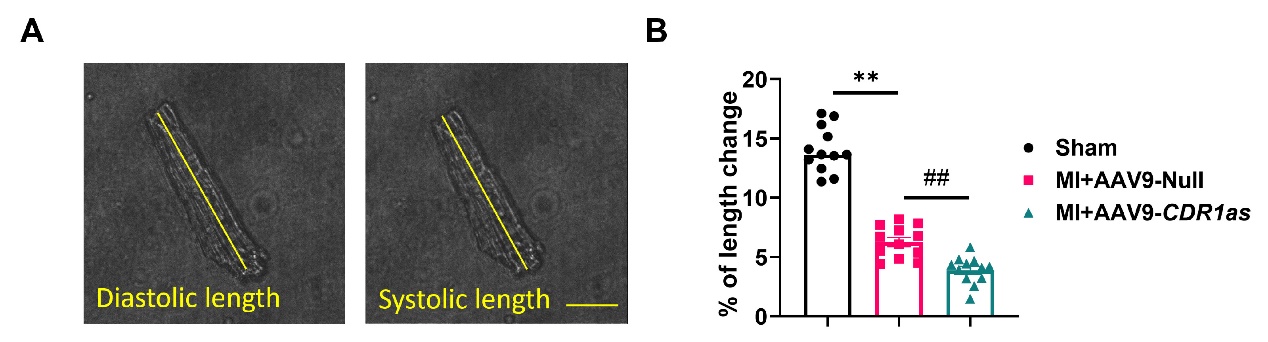
**

**Fig****ure S5. Overexpression of CDR1as impairs cardiac contractility in mice with MI** (A) Changes in the systolic length and diastolic length of isolated single ventricular myocytes. Scale bar: 20 μm. (B) Effect of the overexpression of CDR1as on sarcomere shortening (SS). ^**^ *P* < 0.01 *vs*. Sham; ^##^*P* < 0.01 *vs*. MI+ AAV9-Null; *n*=12. The data are presented as the mean ± SEM.


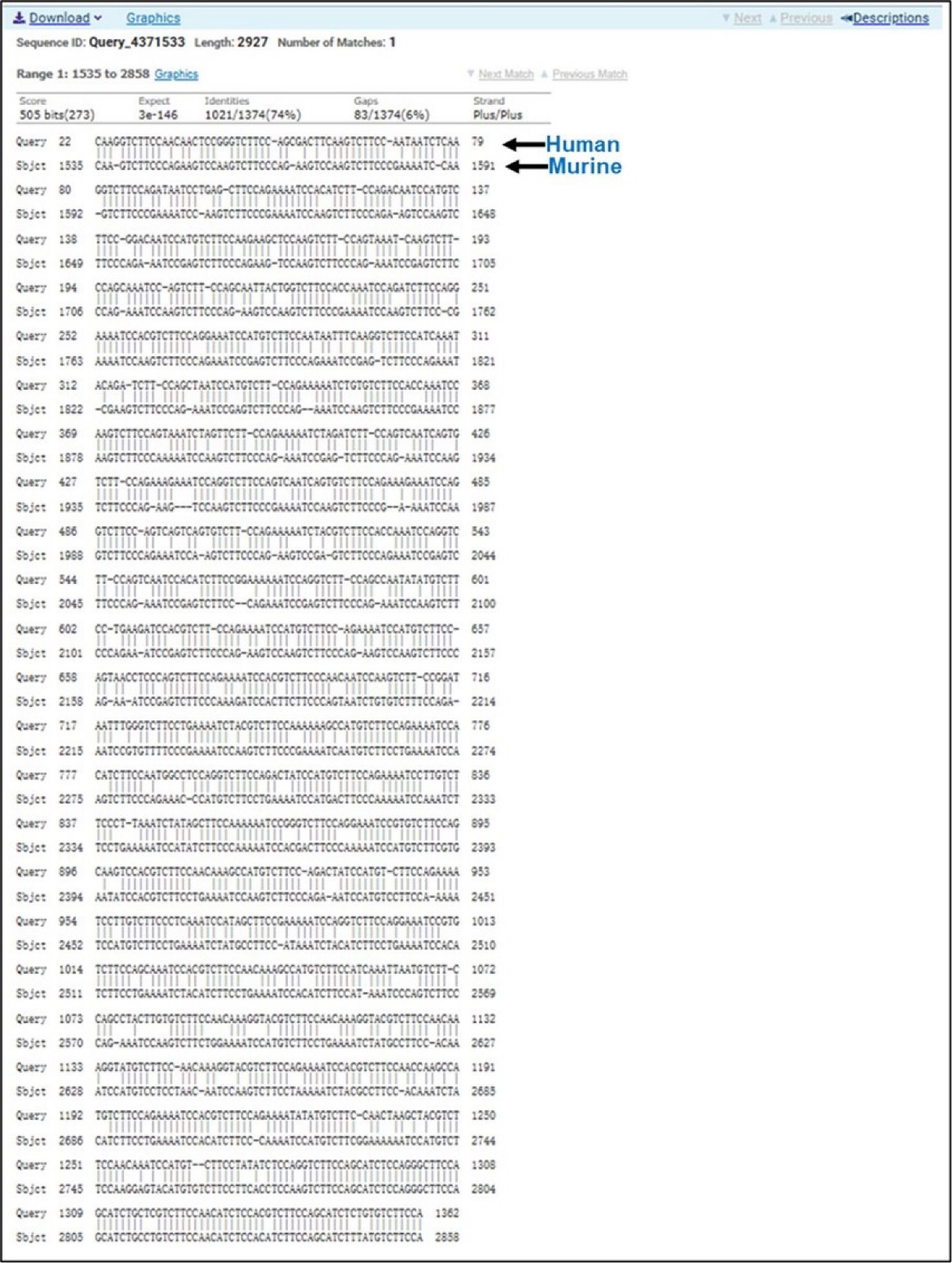


**Figure S6. Alignment of murine circRNA CDR1as and human orthologs**


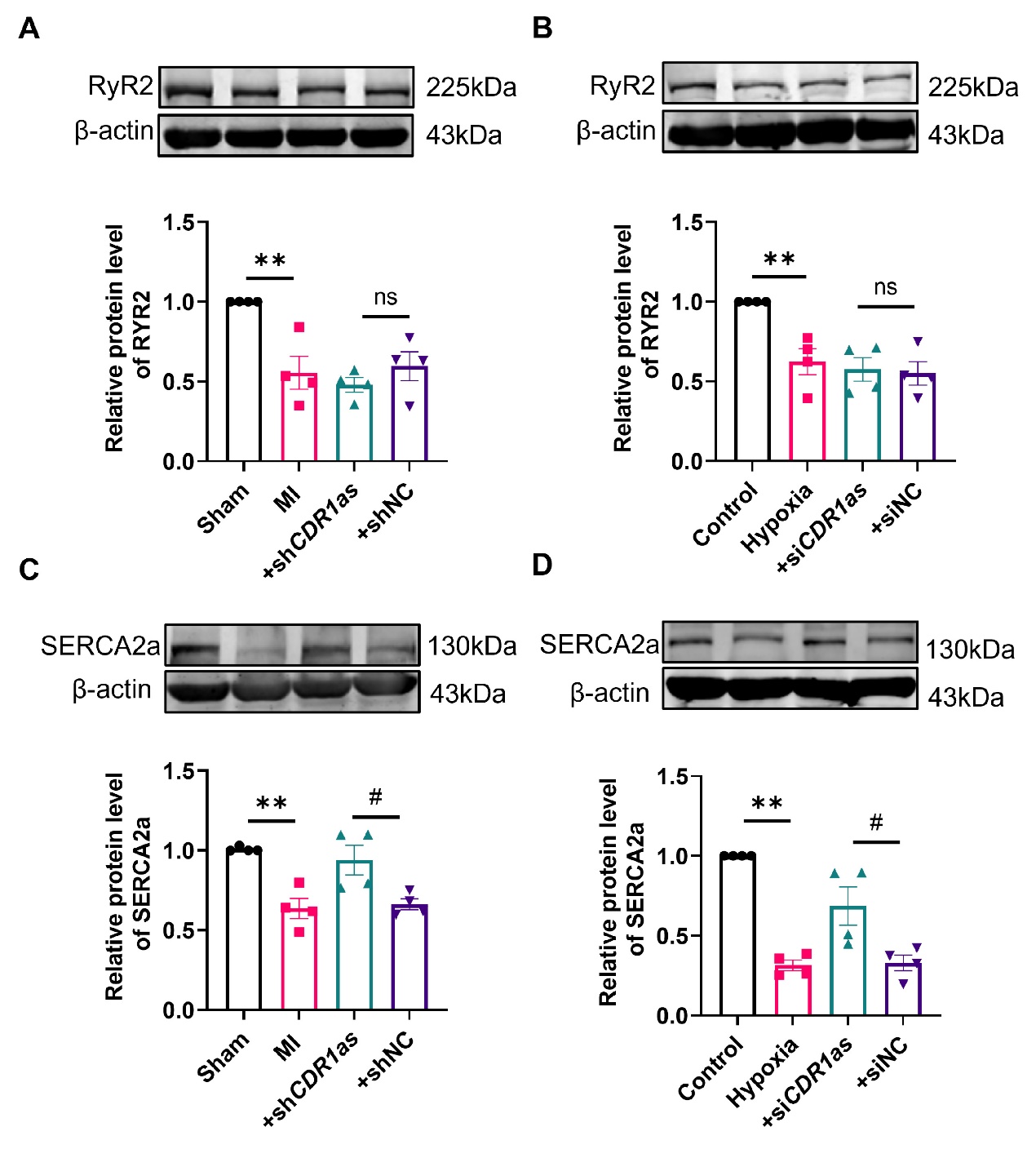


**Figure S7. Effects of CDR1as on the expression of Ca^2+^-handling regulatory proteins other than Cav1.2.** (A) Representative western blots and quantified protein levels of RyR2 in MI mice pretreated with AAV9-shCDR1as. ^**^*P* < 0.01 *vs.* Sham, *P*>0.05 *vs.* + shNC; *n* = 4. (B) The protein level of RyR2 after the silencing of CDR1as in hypoxia-cultured cardiomyocytes. ^**^*P* < 0.01 *vs.* Control, *P* >0.05 *vs.* siNC; *n* = 4. (C) Representative western blots and quantified protein levels of SERCA2a in MI model mice pretreated with AAV9-shCDR1as. ^**^*P* < 0.01 *vs.* Sham, ^#^*P*<0.05 *vs.* + shNC; *n* = 4. (D) The protein level of SERCA2a after the silencing of CDR1as in hypoxia-cultured cardiomyocytes. ^**^*P* < 0.01 *vs.* Control, ^#^*P*<0.05 *vs.* siNC; *n* = 4.
